# Editorial: Heterogeneous catalysts for C1 molecules conversion

**DOI:** 10.3389/fchem.2022.1121871

**Published:** 2023-01-11

**Authors:** Runping Ye, Xusheng Wang, Gang Wang, Yanping Chen, Qinghua Lai, Riguang Zhang

**Affiliations:** ^1^ Key Laboratory of Jiangxi Province for Environment and Energy Catalysis, Institute of Applied Chemistry, School of Chemistry and Chemical Engineering, Nanchang University, Nanchang, China; ^2^ Institute of Functional Porous Materials, School of Materials Science and Engineering, Zhejiang Sci-Tech University, Hangzhou, China; ^3^ Institute for Catalysis, Hokkaido University, Sapporo, Japan; ^4^ State Key Laboratory of Catalysis, Dalian Institute of Chemical Physics, Chinese Academy of Sciences, Dalian, China; ^5^ College of Engineering and Applied Science, University of Wyoming, Laramie, WY, United States; ^6^ State Key Laboratory of Clean and Efficient Coal Utilization, Taiyuan University of Technology, Taiyuan, China

**Keywords:** heterogeneous catalysis, C1 molecules conversion, advances and perspective, carbon dioxide, hydrogen

## Introduction

One-carbon (C1) chemistry is a sustainable, environmentally friendly reaction step for the synthesis of chemicals and fuels, thus it has been an effective approach to solving the depletion of fossil fuels. Specifically, C1 chemistry refers to the synthetic chemistry from the conversion of original compounds with one carbon atom, such as CO, CO_2_, CH_4_, HCHO, HCOOH, and CH_3_OH. The heterogeneous catalysts have been employed for C1 chemistry because of their high conversion efficiency and facile recycling. This editorial work mainly introduces C1 molecules conversion by different catalytic approaches such as electro-, photo-, and thermo-catalytic process.

The key to C1 molecules conversion is to design robust catalysts. A series of conventional thermal catalysts have been constructed and reported for C1 molecules conversion to fuels and chemicals (Jin Z. et al., 2020; Parastaev A. et al., 2022; Rabelo-Neto R. C. et al., 2022). For the sustainable development to produce energy especially hydrogen energy, ethanol, gasoline, and starch, more and more photo-, electro, plasma-, bio-catalysts have been designed for the green conversion of C1 molecules. We have provided a perspective on CO_2_ hydrogenation to C_2+_ products by two typical routes over heterogeneous catalysts (Ye R. P. et al., 2019). As an example, 410 mg/(L·h) of starch could be artificially synthesized within 4 h through 11 core reaction steps from CO_2_ under the combination of thermo- and bio-catalysts (Cai T. et al., 2021). Sonali et al. have also summarized the progress of core-shell structured materials for thermo-, photo, and electro-catalytic conversion of CO_2_ (Das S. et al., 2020). Numerous papers have reported on this Research Topic and the representative advances will be introduced in the next section.

## Significant advances in this Research Topic

Herein we would introduce advances in the collected papers on this Research Topic. Climate change is a global problem and researchers have focused on investigating CO_2_ conversion. However, it is still difficult to develop a robust catalyst for CO_2_ conversion due to its high stability. Compared with traditional oxide-supported catalysts, perovskite-type mixed oxides-based catalysts have attracted more and more attention due to their high CO_2_ activation performance. Wu et al. have provided a review that focuses on emerging perovskite-type mixed oxides–based catalysts for CO_2_ conversion. Three typical transformation reactions of CO_2_ to high-value products on perovskite catalysts have been commented on. The related reaction mechanisms and the reasons for catalyst deactivation were analyzed in detail. Then three strategies were proposed to improve their catalytic performance, including increasing oxygen vacancies, enhancing the active metal dispersion, and tuning the strong metal-support interactions. The specific modified methods were also discussed, including optimizing preparation conditions, adding various additives, introducing appropriate metal substitution and re-loading to other oxide supports, and so on. Finally, future research in this field was provided, aiming to pave the way for net-carbon emission in modern society through sustainable development and efficient utilization of CO_2_.

To relieve the problem of easily sintering and coking of catalysts for dry reforming of CO_2_/CH_4_, Chen et al. investigated the structure and catalytic performance of Ni/SiO_2_ catalysts prepared by three different preparation methods, and pointed out that the Ni/SiO_2_-AE catalyst prepared by ammonia evaporation (AE) method achieved superior catalytic performance than other two preparation methods. This is mainly because that the Ni/SiO_2_-AE catalyst has smaller Ni nanoparticles, strong interaction between metal and supports, as well as abundant Ni-O-Si units. These factors improve its activity and sintering resistance ability. In addition, the Ni-O-Si units as the active sites decomposed methane into CH_x_^*^, avoiding the direct generation of C^*^ and thus greatly enhancing the catalyst carbon resistance. The Ni/SiO_2_-AE catalyst showed good resistance to sintering and carbon deposition during the stability test for 210 h. However, the uneven distribution of nickel species and the large size of Ni nanoparticles in the catalyst prepared by the impregnation method led to poor catalytic performance. The Ni species distribution on the catalysts prepared by the sol-gel method was uniform, but Ni nanoparticles were coated by the silica network, then the effective exposed active sites were reduced, resulting in poor catalyst activity. Therefore, this nice work highlights the influence of the synthetic method on the catalytic properties for C1 molecules conversion.

In addition to CO_2_ conversion, hydrogen production and utilization is another important topic for C1 molecules conversion. Hong et al. have prepared an efficient FeNiOOH/NF (NF = nickel foam) electrocatalyst with hierarchical nanostructures for hydrogen production from water splitting. The hydrogen could also be produced from methanol and formic acid as liquid hydrogen storage carriers due to their stable properties for transportation. The produced hydrogen could be used for CO/CO_2_ hydrogenation to other chemicals like ethanol and gasoline. Thus, the on-site hydrogen production and further hydrogenation reactions are more and more important. Last but not at least, (Yang et al.) have used the metal-organic frameworks as a template to prepare an Ag/ZnO@N-carbon catalyst with superior photocatalytic activity for degradation of RhB, converting 98.65% of RhB after 25 min irradiation. Similarly, the photocatalytic conversion of C1 molecules is also important due to its mild reaction condition.

## Summary and outlook

Nowadays, more and more significant progress through the different catalytic systems has been made for C1 molecules conversion, especially the photo- and electro-catalytic systems for CO_2_/CH_4_ conversion under mild reaction conditions. However, there are still many bottleneck problems to be solved. For example, the severe reaction conditions and related huge energy consumption for thermos-catalytic C1 molecules conversion. However, scaling up and industrialization applications for these emerging photo- and electro-catalysts is still challenging. Although the photo- and electro-catalytic systems are greener and more sustainable, their conversion efficiency is relatively low, and corresponding industrial application is still slow. The development of robust catalysts and advanced equipment would be important to this research field. Moreover, we think the future development direction is the precise design of catalysts with the aid of artificial intelligence under a deep understanding of potential reaction mechanisms. In addition, the dynamic or real structure of catalysts under the reaction conditions should also be revealed with more operando characterization technologies. The study on the C1 chemistry would bring more and more green chemicals and fuels in the future.

## Data Availability

The original contributions presented in the study are included in the article/supplementary material, further inquiries can be directed to the corresponding author.
